# Continuous Ventricular Irrigation for Intraventricular Hemorrhage

**DOI:** 10.1007/s11910-025-01468-w

**Published:** 2025-11-13

**Authors:** Emily G. Dunbar, Tanvika Vegiraju, Andrew P. Carlson

**Affiliations:** 1 Department of Neurosurgery, University of Virginia, 1215 Lee St, Charlottesville, VA 22903 USA; 2 University of Virginia School of Medicine, Charlottesville, VA USA

**Keywords:** Intraventricular hemorrhage, IVH, Active CSF exchange, Continuous irrigation, EVD, IRRAflow

## Abstract

**Purpose of review:**

Despite decades of research, intraventricular hemorrhage (IVH) remains a devastating condition with high morbidity and mortality. Traditional external ventricular drains (EVDs) have long served as the cornerstone of surgical management but are limited by various complications. This review evaluates recent literature on continuous ventricular irrigation as an alternative approach to treating IVH.

**Recent findings:**

Early data surrounding continuous ventricular irrigation systems, including retrospective comparative studies and case series are encouraging. However, existing randomized data are limited by small sample size and methodological flaws. Larger, ongoing studies such as ACTIVE and ARCH aim to provide more definitive evidence.

**Summary:**

Continuous ventricular irrigation offers theoretical and practical advantages over static drainage in IVH patients, including enhanced clot clearance and improved catheter patency, particularly when combined with continuous thrombolytic therapy. Optimized protocols for irrigation rates, medication dosing, and timing are still being investigated. Robust clinical trials are necessary to validate the approach and establish best practices.

## Introduction

Hemorrhagic strokes are associated with high morbidity and mortality [1]. They are subdivided into intracerebral (ICH) and subarachnoid hemorrhages (SAH), both of which can be complicated by intraventricular extension of hemorrhage (IVH), known as secondary IVH. Primary IVH originates solely in the ventricles and is exceedingly rare [2, 3]. Regardless of its origin, IVH is a poor prognostic factor [3, 4].

The underlying pathophysiology resulting in poor outcomes in IVH patients is complex and not yet fully understood. Blood clotting and degradation have been implicated in obstructive hydrocephalus, vasospasm, and delayed cerebral ischemia (DCI) [5]. Current medical management focuses on preventing hemorrhage expansion and monitoring for changes in neurologic exam. Surgical intervention involving the clearance of blood products has been a topic of interest for decades. Proposed solutions historically included cerebrospinal fluid (CSF) diversion, ventricular and cisternal irrigation, thrombolytic administration, and endoscopic clot evacuation. Despite extensive research, no single approach has proven superior to others when comparing long-term functional outcomes. With that said, certain interventions have shown promising safety and survival benefits in selected studies [6–18]– [19].

This review discusses recent literature on continuous ventricular irrigation in IVH patients, with particular focus on historical context and technological advances in IVH management. 

### Where did the Idea of Continuous Irrigation Originate?

Historically, external ventricular drain (EVD) placement has been the preferred surgical intervention for IVH with risk of obstructive hydrocephalus. While effective in diverting CSF, EVDs are prone to complications [18, 20, 21]. The catheters often become occluded by the same blood products that block the ventricular outflow. This can result in inadequate CSF drainage and elevated intracranial pressures (ICPs). Clearing the occlusion requires either flushing the catheter, which violates the sterile circuit, or completely replacing it. Both interventions increase the risk of infection and impose additional risk and cost [21].

Given their prevalence, multiple studies closely examined EVD complications. An international retrospective analysis of 401 EVD patients reported complication rates as high as 56%, with infection (24%), obstruction (17%), and early catheter replacement (12%) being the most common issues [18]. Permanent EVD occlusion was 3.4 times more likely in smaller-diameter catheters and 6.7 times more likely in patients who were therapeutically anticoagulated prior to IVH, based on a prospective observational study examining data from 98 patients [20]. The same authors noted that catheter replacement was associated with new intracranial hemorrhage in 62% of cases, though only 2% resulted in neurological symptoms. Interestingly, the mode of EVD drainage may also affect obstruction incidence. According to a 60-patient randomized controlled trial (RCT), intermittent drainage via EVD is associated with lower occlusion and related complication rates compared to continuous drainage (23.1% versus 52.9%) [21].

Several devices have been designed to address these issues [22]. Antibiotic-impregnated catheters have been shown to decrease infection risk and have become common practice [23]. Larger-bore EVD catheters with specialized fenestration patterns and antithrombotic coatings, such as the CerebroFlo, are also being trialed and show promise in reducing occlusions [20, 24]. Though exciting, these advances aid only passive drainage.

One of the earliest standardized protocols for IVH irrigation targeting obstruction prevention and efficient hemorrhage clearance originated in the neonatal literature. The Drainage, Irrigation and Fibrinolytic Therapy (DRIFT) trial investigated whether thrombolytic irrigation could improve outcomes in premature infants with IVH [6]. IVH is a common and crippling complication of prematurity. Nearly 50% of grade IV IVH infants experience severe cognitive disability and become shunt dependent, largely due to posthemorrhagic ventricular dilatation (PHVD) [25]. PHVD has been associated with early hemorrhagic infarction of periventricular white matter as well as progressive global injury of the developing cerebral tissue. The underlying pathophysiology is thought to be similar to that of IVH sequelae in adults, making DRIFT a study of interest not just for the premature infant population.

In the DRIFT protocol, patients received a single dose of intraventricular recombinant tissue plasminogen activator (rtPA) followed by artificial CSF irrigation. Irrigation inflow was via a frontal catheter, with outflow through a posterior, occipital horn catheter [6]. Although the intervention increased rates of secondary intraventricular bleeding, it significantly reduced severe cognitive disability at 2- and 10-year follow-ups [6, 26]. Unfortunately, improvements in concrete clinical endpoints such as motor function, shunt dependency, and mortality were not observed. Given the technical complexity of the DRIFT protocol and uncertain benefit, the approach has not been widely adopted in most neonatal intensive care units. However, this trial, among others, did prompt investigators to explore the efficacy of a less expensive thrombolytic, urokinase. They found that urokinase may decrease inflammation and neurotoxicity compared to rtPA in infants [27]. Despite these findings, urokinase is not currently available for use in the United States, though it remains an option in low-resource settings.

The DRIFT trial also contributed to the rising interest surrounding thrombolytic use in adult IVH patients, with promising results noted in several studies. Phase III of Clot Lysis Evaluation of Accelerated Resolution of Intraventricular Hemorrhage (CLEAR III) was a large multicenter, double-blinded RCT that evaluated the efficacy of recurrent intraventricular rtPA administration *without irrigation* in clearing obstructive IVH [7]. The authors found that the rtPA group experienced fewer serious adverse events and lower mortality compared to the normal saline control group. Unfortunately, rtPA did not improve functional outcomes. A prespecified volume threshold analysis found that post-treatment residual IVH volume ≤ 15% was independently associated with improved outcomes, suggesting that volume reduction, not just ventricular outflow clearance, may be an important treatment goal [7, 28]. A secondary analysis of the CLEAR III data interestingly found that, unlike in cases of hydrocephalus caused solely by IVH, rtPA was not effective in patients with large thalamic hemorrhages exerting indirect mass effect on the third ventricle [29]. Therefore, the latter patient group may benefit from alternative treatment approaches (e.g., direct clot evacuation), as suggested by subsequent studies [30].

Aside from thrombolytic ventricular irrigation, cisternal irrigation and endoscopic clot evacuation have also been explored as alternative approaches to reduce hemorrhagic burden. Cisternal irrigation requires the performance of ventriculocisternostomy, which can be achieved either stereotactically or via an open approach in patients undergoing aneurysmal clipping. Despite the additional technical complexity and potential for complications, existing literature reports improved neurologic outcomes, with decreased rates of vasospasm and DCI [31–34]. Endoscopic IVH evacuation utilizes intraprocedural active irrigation. Proposed benefits of endoscopic irrigation include the direct visualization of both the hematoma and post-evacuation EVD. Hematoma visualization translates into more efficient, rapid evacuation with decreased risk of rebleeding, while non-blind EVD insertion ensures proper placement and minimizes risk of choroid plexus injury. Multiple studies assessing the impact of these benefits on clinical outcomes have been published over recent years [9–13, 35]. A network meta-analysis, which included 16 publications, found that endoscopic washout was superior to EVD alone or EVD with fibrinolytics in terms of survival, functional outcomes, and reduced complications such as rebleeding, infection, and shunt dependence [9]. Although these findings have been corroborated by additional studies [10–13], the overall strength of evidence is limited by small sample sizes and lack of extensive RCTs. To address this issue, a large prospective, multi-center RCT is currently underway comparing endoscopic IVH evacuation to EVD drainage, with the primary endpoint being 12-month survival [35].

## Modern Continuous Irrigation

Historical approaches to irrigation typically rely on accurate leveling and patency of the outflow catheter. Therefore, these systems are prone to technical failure and harmful complications, especially over-infusion. Given the low and fixed intracranial volume (i.e., Monro-Kellie doctrine), over-infusion of irrigant without a functional outflow tract or ICP-based failsafe mechanism can quickly lead to herniation and death. Consequently, continuous irrigation systems must be closely monitored and ideally incorporate real-time ICP feedback.

The IRRAflow device represents a substantial advancement in IVH management. It is a closed-loop system with a dual-lumen catheter that enables controlled, continuous intraventricular irrigation with simultaneous ICP monitoring [36]. Real-time pressure monitoring enables timely irrigation and drainage setting adjustment, minimizing the risk of harmful ICP fluctuations that can occur during irrigation. Since the system remains closed, unlike conventional EVD circuits that must be opened for irrigation and medication administration, the infection risk is substantially reduced.

Studies assessing the safety and efficacy of continuous irrigation have had early, promising results. Several small retrospective studies found continuous irrigation to promote faster ventricular outflow tract clearance, decreased EVD duration and replacement rates, and more favorable functional outcomes [14, 15, 17, 19]. These findings were supported by our 21-patient case-control study, which showed better overall outcomes with IRRAflow-mediated rtPA irrigation, as well as by an international case-control study with 519 patients (118 IRRAflow, 401 EVD) [16, 18]. The larger study analyzed a variety of conditions requiring a ventricular drain, finding generally lower complication rates and improved functional outcomes with the continuous irrigation system. The rates of insertional hemorrhage and inadvertent catheter removal did not vary between the two groups. Though more than half of the patients studied had IVH, subgroup analysis of this population was not presented, limiting the utility of the findings. Challenging the above-noted observations is a small RCT comparing IRRAflow to standard EVD. The trial had to be stopped early due to increased IRRAflow complications, mainly catheter displacements and infections [37]. It is important to note that this study had significant methodological limitations. The clinical team routinely utilized bolt-based EVDs and lacked experience with tunneled catheter systems, leading to inadequate catheter securing and increased risk of displacement. Most centers in the United States routinely use tunneled catheters, making this concern less generalizable. Additionally, infection risk in the IRRAflow group may have been exacerbated by the use of multiple drains per patient and re-advancement of displaced tunneled catheters through the existing subcutaneous tracks. Despite these issues, prior to the early termination of the trial, there was a trend toward better outcomes in the IRRAflow group, with mortality rate of 27% versus 40% in the control group [37].

The above results underscore the importance of well-designed trials and standardized protocols when evaluating new technologies. To that end, IRRAS, the manufacturer of IRRAflow, is sponsoring two multicenter, prospective RCTs:

- ACTIVE (Use of Active Fluid Exchange to Treat Intraventricular Hemorrhage): This study hypothesizes that active irrigation will reduce rates of catheter occlusion and infection. The investigators also plan to evaluate overall blood clearance, required treatment time, and long-term patient outcomes [38].
- ARCH (Active Removal of IntraCerebral Hematoma via Active Irrigation of the Ventricular System): This study assesses the impact of rtPA irrigation on hematoma clearance time and patient outcomes [39].

These trials along with several nascent investigator-initiated studies are expected to provide critical evidence that will define the role of continuous irrigation in the management of IVH.

## Institutional Experience and Perspective

At our institution, the implementation of the IRRAflow continuous irrigation system was relatively straightforward with the support of a physician “champion” who guided clinical integration. We encountered a high level of enthusiasm from nursing staff and families alike, largely due to the visible removal and clearing of bloody CSF, which reinforced the perception of active treatment.

Initial challenges primarily involved gaining familiarity with the device and its specific nuances compared to traditional EVD systems. For example, IRRAflow catheters cannot be flushed manually through the pressure-sensing cartridge, which differs from conventional drainage systems. Flushing must be done by the system itself, which not only maintains sterility but also allows for concurrent ICP monitoring. In addition, to ensure consistent practice and minimize the risk of over- or under-drainage/infusion, all IRRAflow parameters (e.g., bag height, treat above setting) must use the same measurement units. We standardized our measurements to “mmHg,” the gold standard for pressure monitoring. Since using cmH₂O for traditional EVD bag height and mmHg for ICP measurement is a common practice, new IRRAflow adopters must be aware of this nuance and plan accordingly to avoid confusion and related complications. Of note, in complex clinical scenarios, we observed occasional discrepancies between the pressure reported by the IRRAflow catheter, which is measured at the catheter tip, and parenchymal monitors. These differences likely reflect local pressure gradients, as previously described by others, and may occur for a variety of reasons [40]. In cases where there is concern about the accuracy of ICP readings, placement of a parenchymal ICP monitor may be warranted to ensure that the IRRAflow device can safely continue to irrigate. Lastly, a frequently encountered concern worth mentioning is the occurrence of net positive irrigation, where inflow volume exceeds outflow. While this warrants imaging to exclude over-infusion, we have found it is often a benign phenomenon, particularly when it coincides with improved ventricular clearance. We suspect this may reflect restoration of physiologic CSF/irrigant absorption through arachnoid granulations once obstruction resolves. In the absence of ICP elevation or clinical deterioration, such mismatches are typically well tolerated.

Currently, our standard practice is to initiate passive drainage immediately following IRRAflow insertion to relieve increased ICPs. Irrigation is only started following a confirmatory computed tomography (CT) scan verifying proper catheter placement. If thrombolytic therapy is planned, rtPA irrigation typically begins as soon as hematoma stability is confirmed on repeat imaging. When there is a clear asymmetry in clot burden, we target catheter placement to the side with greater hemorrhage to maximize clot removal. In our experience, patients treated with continuous irrigation via IRRAflow had rapid IVH resolution, most achieving near complete clearance of the ventricular outflow tract within 72 h of irrigation system insertion. These results are consistent with the case-control study discussed earlier [16]. Below, we present several cases of patients with significant initial IVH and subsequent clearance.

### Case 1: PICA Aneurysm Rupture with Casting of the Fourth Ventricle and Cisterna Magna

**Fig. 1:**
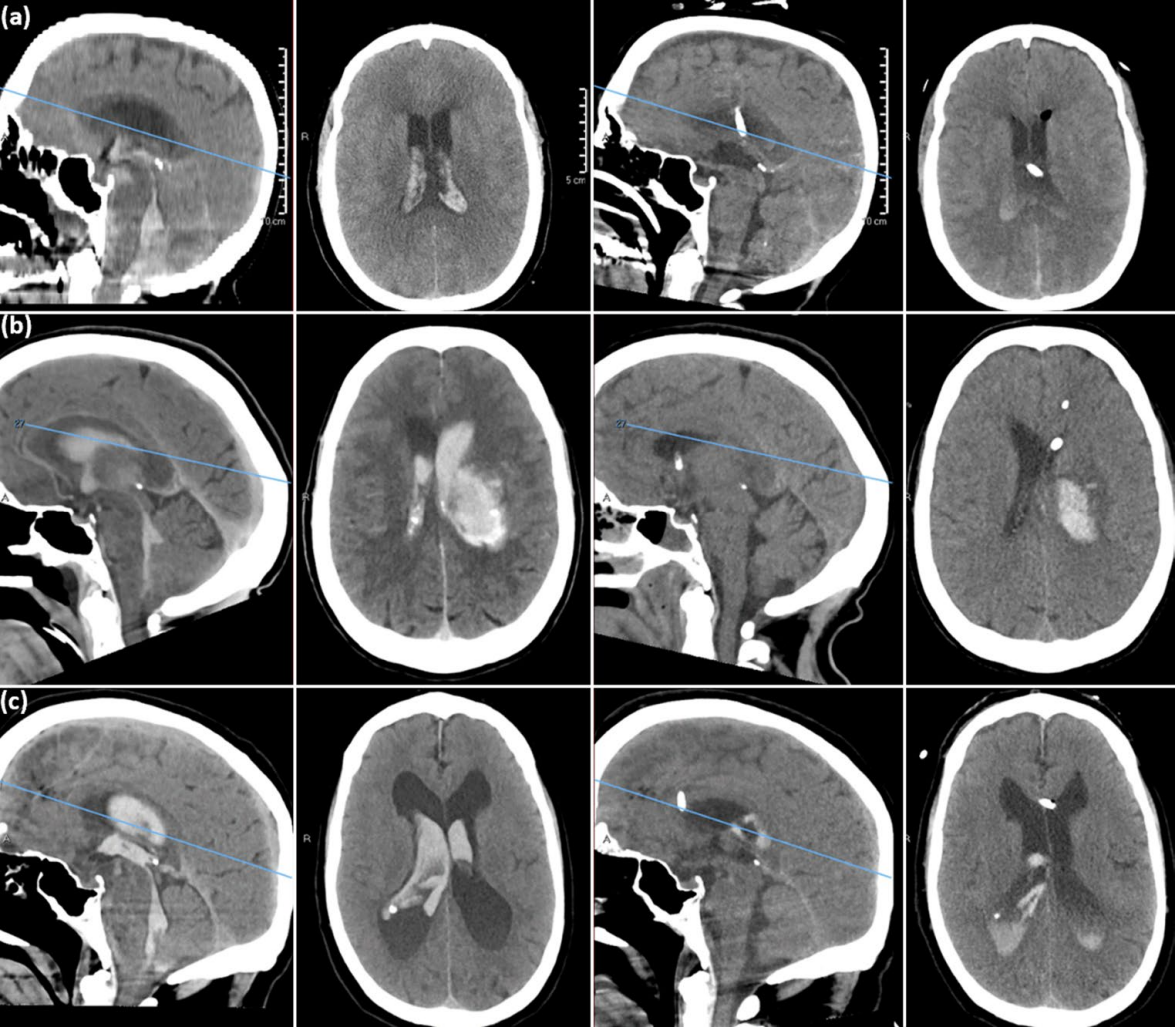
Sagittal and axial computed tomography (CT) images on admission (left) versus 72 h later (right). (**a**) case 1: PICA aneurysm rupture with casting of the fourth ventricle and cisterna magna; (**b**) case 2: Thalamic hemorrhage with ventricular casting; (**c**) case 3: IVH from choroidal aneurysm in moyamoya disease

This 68-year-old female presented with an HH grade 5, mFS grade 4 SAH in the setting of a ruptured fusiform distal PICA aneurysm related to a small cerebellar AVM. IRRAflow was placed on admission, and after securing the aneurysm, irrigation with rtPA at 30 mL/hr was initiated. Our standard concentration of rtPA is 2 mg in 500 mL of normal saline, which results in approximately 1 mg of rtPA being delivered every 8 h of continuous irrigation, similar to the MISTIE III and CLEAR III trials [7, 8]. The rtPA irrigation continued for approximately 36 h, following which a repeat scan (Fig. 1a) showed significant clearance of intraventricular blood, particularly around the foramen magnum. The IRRAflow remained in place through the DCI window.

### Case 2: Thalamic Hemorrhage with Ventricular Casting

This 64-year-old female presented with a large left basal ganglia ICH with intraventricular extension and casting of the left lateral ventricle. A left frontal, IRRAflow was placed freehand at bedside on admission, with catheter positioning in the left frontal horn confirmed via CT head. Active fluid exchange with LR was started at 30 mL/hr. On hospital day one, the patient was transitioned to rtPA irrigation for 24 h at approximately 1 mg every 8 h, and then reverted to LR once the third and fourth ventricles were cleared. Imaging at 72 h (Fig. 1b) showed near-complete clearance of the remainder of the ventricular system. IRRAflow was subsequently removed on hospital day five.

### Case 3: IVH from Choroidal Aneurysm in Moyamoya Disease

This 64-year-old male presented with headache and altered mental status. He was found to have severe hydrocephalus and vascular abnormalities consistent with right-sided moyamoya disease. A choroidal pseudoaneurysm was confirmed on angiography as the source of the hemorrhage. A right frontal IRRAflow was placed with standard irrigation of LR at 30 mL/hr. Due to the location of the hemorrhage in the temporal horn, rtPA was initiated cautiously with close imaging surveillance. Approximately 48 h of irrigation resulted in complete clearance of the ventricular outflow tract and reduction in ventricular size (Fig. 1c). The IRRAflow was removed and replaced by a lumbar drain.

#### Abbreviations used

Hunt-Hess (HH), modified Fisher Scale (mFS), subarachnoid hemorrhage (SAH), posterior inferior cerebellar artery (PICA), arteriovenous malformation (AVM), recombinant tissue plasminogen activator (rtPA), delayed cerebral ischemia (DCI), intracerebral hemorrhage (ICH), computed tomography (CT), Lactate Ringer’s (LR).

## Conclusion

IVH remains a devastating complication of both intracerebral and subarachnoid hemorrhages, with high rates of morbidity and mortality despite decades of investigation into optimal management strategies. While traditional EVDs have long served as the standard of care for CSF diversion and ICP control, their limitations, namely catheter occlusion, infection, and suboptimal clearance of blood products, have prompted exploration of alternative and adjunctive therapies. Interventions such as thrombolytic irrigation, cisternal washout, and endoscopic evacuation have demonstrated some benefit in terms of clot clearance and survival. However, evidence supporting improvement in long-term functional outcomes remains inconsistent, and widespread adoption has been limited by technical complexity and procedural risks.

Continuous intraventricular irrigation systems such as IRRAflow represent a novel and promising therapeutic strategy. These devices combine closed-loop CSF exchange with real-time intracranial pressure monitoring, potentially offering advantages in safety and efficacy. Early studies report faster hemorrhage clearance, reduced catheter obstruction and infection rates, as well as potential functional benefits; however, further data are needed.

Ongoing large-scale, multicenter randomized trials, such as ACTIVE and ARCH, will be critical in determining the efficacy and safety of continuous irrigation, both with and without adjunctive thrombolytic therapy. These studies will also help establish standardized protocols for irrigation rates, drainage thresholds, choice of irrigation fluid, and thrombolytic dosing. Additionally, integration of continuous irrigation systems into broader minimally invasive hemorrhage evacuation strategies, such as those used in CLEAR III and MISTIE III, may offer further insights into their role in comprehensive hemorrhagic stroke management.

While our institutional experience with IRRAflow has been encouraging, successful implementation involves a learning curve and requires interdisciplinary collaboration and attention to device-specific nuances. With continued innovation and more robust evidence, continuous irrigation has the potential to meaningfully improve patient outcomes and change the future of IVH management. 

## Data Availability

No datasets were generated or analysed during the current study.

## Key References

- Carrera DA, Mabray MC, Torbey MT, Andrada JE, Nelson DE, Sarangarm P, et al. Continuous irrigation with thrombolytics for intraventricular hemorrhage: case-control study. Neurosurg Rev. 2024;47(1):40. 10.1007/s10143-023-02270-3.
  - A case-control study showing that IRRAflow tPA irrigation is associated with decreased clot clearance time and ICU length of stay, as well as improved functional outcomes at 90-day follow-up.

- Jahromi BR, Monachello G, Khan MB, Hodge JO, Garavaglia J, Turner RC, et al. Active Cerebrospinal Fluid Exchange vs. External Ventricular Drainage in the Neurocritical Care Unit: An International, Retrospective Cohort Study. Neurosurgery. 2025 [Preprint]. doi:10.1227/neu.0000000000003587.
  - A large international case-control study finding that IRRAflow is associated with lower complication rates and improved clinical outcomes.

- Whitelaw A, Jary S, Kmita G, Wroblewska J, Musialik-Swietlinska E, Mandera M, et al. Randomized trial of drainage, irrigation and fibrinolytic therapy for premature infants with posthemorrhagic ventricular dilatation: Developmental outcome at 2 years. *Pediatrics.* 2010;125(4):e852–8. 10.1542/peds.2009-1960.
  - A randomized trial demonstrating that thrombolytic irrigation in premature infants with IVH reduced mortality and severe cognitive disability at two-year follow-up.

- Hanley DF, Lane K, McBee N, Ziai W, Tuhrim S, Lees KR, et al. Thrombolytic removal of intraventricular haemorrhage in treatment of severe stroke: results of the randomised, multicentre, multiregion, placebo-controlled CLEAR III trial. *Lancet.* 2017;389(10069):603–11. 10.1016/S0140-6736(16)32410-2.
  - A double-blinded randomized controlled trial finding that intraventricular irrigation with rtPA decreased adverse events and mortality, but did not improve functional outcomes.
